# A rare case of alirocumab (PCSK9 inhibitor)-associated acute interstitial nephritis 

**DOI:** 10.5414/CNCS111443

**Published:** 2024-09-16

**Authors:** Nazia Habib, Chase Yuan, Andrea Lightle, Mauricio Monrroy, Swati Mehta

**Affiliations:** 1 Department of Medicine, Division of Nephrology, Albany Medical Center,; 2 Albany Medical College, and; 3 Department of Pathology, Albany Medical Center, Albany, NY, USA

**Keywords:** PCSK9, alirocumab, acute interstitial nephritis, kidney biopsy

## Abstract

Acute interstitial nephritis (AIN) is a common cause of hospital-acquired acute kidney injury (AKI) [1]. The most common cause of AIN is drug-induced AIN, which accounts for 60 – 70% of cases [2]. Alirocumab, a proprotein convertase subtilisin/kexin type 9 inhibitor (PCSK9 inhibitor) is a monoclonal antibody that lowers low-density lipoprotein-C levels by inhibiting the PCSK9 protein [3]. Common adverse events reported with alirocumab include injection-site reactions, myalgia, neurocognitive disorders, and ophthalmologic disorders [4]. There is paucity of data, with few reported cases of AKI, mostly in the form of acute tubular necrosis (ATN) associated with alirocumab [5]. In this article, we present a novel case of AIN associated with the use of alirocumab.

## Case report 

A 57-year-old male with a past medical history of recurrent *C. difficile* colitis, alcohol use disorder, hypertension, hyperlipidemia, and coronary artery disease presented with 5-month history of fatigue and a more recent 3-week history of increasing confusion, vomiting, diarrhea, and malaise. He also endorsed decreased appetite and fluid intake with concomitant oliguria. 

His current prescribed medications included ramipril, propranolol, aspirin, and alirocumab. The patient started once monthly alirocumab as part of a lipid-lowering therapy regimen 8 months ago, and he had taken 3 doses in total. The patient had been on rest of the meds for much longer. He denied taking non-steroidal anti-inflammatory drugs, proton pump inhibitors, recent antibiotics, and diuretics. His last episode of *C. difficile* colitis was 9 months back when he took fidaxomicin. The patient had a history of heavy alcohol use (drank 4 vodkas a night). 

Upon admission to the hospital, complete metabolic panel revealed sodium 133 mEq/L (135 – 145 mEq/L), potassium 4.5 mEq/L (3.4 – 5.2 mEq/L), CO_2_ 12 mmol/L (21 – 30 mmol/L), BUN 110 mg/dL (7 – 22 mg/dL), creatinine 12.52 mg/dL (0.8 – 1.4 mg/L), baseline creatine 0.7, 6 months ago, albumin 3.8 g/dL (3.5 – 5.2 g/dL), calcium 7.8 mg/dL (8.6 – 10.3 mg/L), phosphorus 8.3 mg/dL (2.4 – 4.7 mg/L). Other values were within normal limits. CBC revealed hemoglobin of 7.8 g/dL (13.6 – 16.7 g/dL), white blood cell count was 6.3*10^3/uL (4.0 – 9.0*10^3/uL) and platelet of 206*10^3/uL [130 – 350*10^3/uL] with an unremarkable differential (eosinophils 1%) and CK level 65 IU/L. Urinalysis revealed 3+ hematuria, +1 proteinuria, 0 – 5 WBCs, and coarse granular casts. On stool testing, *C. difficile* gene was positive but negative for toxin presence. 

**Figure 1:**
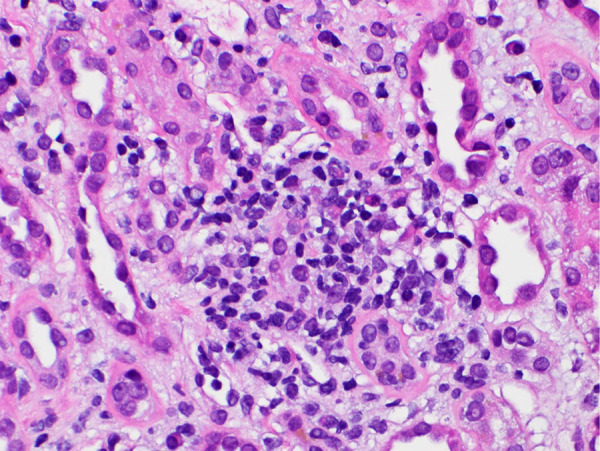
Renal biopsy showed diffuse interstitial edema and mononuclear inflammatory cell infiltrates with foci of tubulitis. The glomeruli are unremarkable. Magnification, × 100.

**Figure 2:**
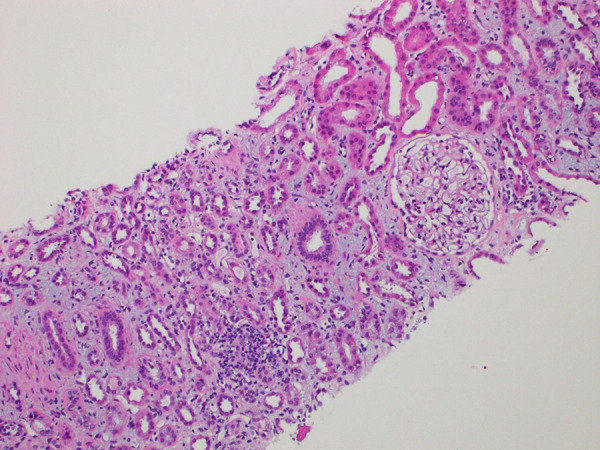
The inflammatory infiltrates are composed predominantly of lymphocytes and histiocytes with fewer plasma cells. Eosinophils (not seen in this image) are rare. Magnification, × 400.

Evaluation for causes of acute kidney injury (AKI) revealed no significant positive findings. Serological workup including hepatitis B, C, anti-neutrophil cytoplasmic antibody, complement levels, double-stranded DNA, and serum-free light chains was negative. On day 5 of admission, the decision was made to proceed with kidney biopsy. The patient underwent kidney biopsy to determine the cause of AKI. Kidney biopsy was consistent with acute interstitial nephritis (AIN) (Figures 1, Figure 2). Other biopsy findings were acute tubular injury with myoglobin cast, consistent with rhabdomyolysis, moderate interstitial fibrosis, and tubular atrophy. 

The patient’s AIN was favored to be medication related, likely PCSK9 inhibitor alirocumab, as this was the only new medication introduced within the few months before his symptoms started. Absence of granulomas and IgG4-positive plasma cells on renal biopsy makes sarcoidosis and IgG4 disease-related AIN less likely. Idiopathic AIN as part of tubulointerstitial nephritis and uveitis (TINU) syndrome was ruled out as the patient did not endorse uveitis. 

The patient’s acute tubular necrosis (ATN) was attributed to rhabdomyolysis from heavy alcohol use. His CPK levels were within normal limits although they were checked 1 week after admission. 

The patient had a combination of drug-induced AIN (focal infiltrate, edema, and tubulitis in renal biopsy) and myoglobin-induced ATN (3+ hematuria without RBCs in urine sediment) to explain the AKI. The patient’s AIN was likely related to PCSK9 inhibitor alirocumab, and steroid treatment was initiated within 1 week of his admission. 

Alirocumab was discontinued. The patient was started on prednisone 60 mg daily for 14 days followed by taper (decreased dose by 10 mg every 5 days until a dose of 10 mg was reached followed by decrease to 5 × 1 week followed by 2.5 × 1 week, then stop). He received a total of 2 sessions of hemodialysis. He was also treated with oral vancomycin for *C. difficile*. Creatinine slowly trended down to 1.8 mg/dL on discharge and was 1.5 mg/dL at week 6 in clinic follow-up. 

## Discussion 

Alirocumab is second-line medication prescribed for the treatment of hypercholesterolemia [6]. The currently known side effects predominantly include pruritis, angioedema, elevated hepatic enzymes, and confusion [4]. The potential renal side effects of the medication have not been well documented except in the form of case reports showing a possible relationship between alirocumab and ATN [5, 7]. In both cases, kidney function returned to baseline after cessation of alirocumab. Our patient likely had a combination of drug-induced AIN from alirocumab use and myoglobin-induced ATN to explain the AKI. To our knowledge, our case represents the first case of AIN associated with alirocumab. While alirocumab has been reported to cause ATN in two case reports [5, 7], none of them reported myoglobin-induced ATN. Whether there is an association between alirocumab and rhabdomyolysis remains unclear to date. In our case, ATN was attributed to rhabdomyolysis from heavy alcohol use. 

Drug-induced AIN is a delayed T-cell mediated hypersensitivity reaction. While the underlying mechanism of alirocumab causing AIN is unclear, its core structure is possibly inciting the immune response either via direct interaction with an allele or receptor or combining with a self-protein to create a hapten [8]. Additional research is required to further elucidate the association between the two. 

Clinical manifestations of AIN may present with non-specific signs and symptoms of AKI. These may include nausea, vomiting, and malaise. However, the classic triad of fever, rash, and eosinophilia is only present in ~ 10% of the patients [9]. Oliguria, although not very common, may be present in patients with AIN like in our case. In a retrospective analysis of 60 cases of AIN, oliguria was present amongst 51% of cases [9]. Furthermore, patients typically have WBCs or WBC casts in urine sediment. Our patient had very few WBCs but no WBC casts, possibly being one of the few cases to have no specific urinary sediment findings [10]. The absence of above urinary findings does not exclude AIN. 

High vigilance and thorough investigations are required to identify AIN as a cause of renal failure. Renal biopsy remains the gold standard in the diagnosis of AIN. Discontinuation of offending drug and early use of glucocorticoid therapy remains the mainstay of treatment [11, 12]. 

## Conclusion 

This case highlights alirocumab as a potential cause of AIN. Clinicians should have high vigilance and do thorough investigations to identify the cause of renal failure in patients on alirocumab. Renal biopsy remains the gold standard to diagnose AIN. Discontinuation of the drug and early use of glucocorticoids usually portend a good prognosis with renal recovery. 

## Authors’ contributions 

Dr. Nazia Habib and Chase Juan contributed in data collection on the patient and drafting of the manuscript. Drs. Monrroy and Mehta also helped in drafting the manuscript and reviewed it once completed. Dr. Lightle helped with pathology images. 

## Funding 

The authors received no financial support for the authorship of this article. 

## Conflict of interest 

The authors declared no conflicts of interest with respect to the publication and authorship of this article.
